# Hydrocarboxylic acid receptor 1 in BAT regulates glucose uptake in mice fed a high-fat diet

**DOI:** 10.1371/journal.pone.0228320

**Published:** 2020-01-30

**Authors:** Eunjin Kwon, Taesik Yoo, Hye-Young Joung, Young-Hwan Jo

**Affiliations:** 1 Division of Endocrinology, Department of Medicine, Albert Einstein College of Medicine, Bronx, New York, Unites States of America; 2 Fleischer Institute for Diabetes and Metabolism, Albert Einstein College of Medicine, Bronx, New York, Unites States of America; 3 Department of Molecular Pharmacology, Albert Einstein College of Medicine, Bronx, New York, Unites States of America; East Tennessee State University, UNITED STATES

## Abstract

Interscapular brown adipose tissue (BAT) has the capability to take up glucose from the circulation. Despite the important role of BAT in the control of glucose homeostasis, the metabolic fate and function of glucose in BAT remain elusive as there is clear dissociation between glucose uptake and BAT thermogenesis. Interestingly, intracellular glycolysis and lactate production appear to be required for glucose uptake by BAT. Here, we specifically examine whether activation of lactate receptors in BAT plays a key role in regulating glucose homeostasis in mice fed a high-fat diet (HFD). When C57BL/6J mice are given HFD for 5 weeks at 28°C, male, but not female, mice gain body weight and develop hyperglycemia. Importantly, high-fat feeding upregulates expression of the lactate receptor hydroxycarboxylic acid receptor 1 (HCAR1) in female C57BL/6J mice, whereas male C57BL/6J mice show reduced HCAR1 expression in BAT. Treatment with the HCAR1 agonist lowers systemic glucose levels in male DIO mice. This reduction is associated with increased glucose uptake in BAT. Therefore, our results suggest that HCAR1 in BAT may contribute to the development of hyperglycemia in male C57BL/6J DIO mice.

## Introduction

Interscapular brown adipose tissue (BAT) is a principal site of nonshivering thermogenesis, which results from the uncoupling of mitochondrial oxidative respiration from ATP production to generate heat [1–3]. This uncoupling protein 1 (UCP1)-dependent thermogenesis is largely fueled by fatty acids from intracellular triglycerides in rodents and humans [4–6]. Additionally, BAT is able to take up glucose from the circulation [2, 7–9]. Indeed, we recently demonstrate that optogenetic activation of sympathetic nerves exclusively innervating BAT promotes glucose uptake, resulting in a rapid reduction in blood glucose levels [10]. Interestingly, it appears that glucose does not contribute to BAT thermogenesis. In fact, only a small portion of glucose taken up is used for thermogenesis in rodents [11, 12]. In addition, there is clear dissociation between glucose uptake and nonshivering thermogenesis in humans [13, 14]. Hence, these prior findings, including our own raise an important question as to the metabolic fate and function of the glucose entering the BAT.

It has been described that lactate production accounts for a large proportion of glucose uptake by BAT following treatment with noradrenaline in rodents [11]. Activation of the beta 3 adrenergic receptor (β3AR) in BAT converts glucose to lactate in BAT [15, 16]. We further shows that optogenetic stimulation of sympathetic nerves innervating BAT increases expression of the lactate dehydrogenase A (*Ldha*) gene [10], consistent with a prior transcriptomic study of BAT showing cold-induced upregulation in *Ldha* expression in BAT [17]. Importantly, inhibition of LDHA blocks the ability of BAT to uptake glucose [10]. Hence, lactate production in BAT appears to be required for glucose uptake. A recent human study demonstrates substantial glucose uptake and lactate release from BAT during warm conditions [6], suggesting that there is an autocrine and/or paracrine release of lactate from BAT. As BAT is a primary organ that expresses lactate receptors [18–21], it is highly plausible that lactate receptors in BAT may detect, sense, and respond to changes in circulating and/or local lactate levels.

BAT expresses the hydrocarboxylic acid receptor 1 (HCAR1) (also known as GPR81) in both rodents and humans [6, 19, 20]. HCAR1 is coupled to G_i/o_ proteins and is activated by lactate [19, 20]. These receptors are primarily expressed in white and brown adipocytes [18–21]. Activation of HCAR1 by lactate inhibits lipolysis in adipocytes of humans, mice, and rats [19, 20, 22, 23]. Importantly, the locally released lactate from BAT, but not from the circulation, inhibits lipolysis when glucose levels are elevated [20]. In this study, we specifically examined whether HCAR1 activation in BAT plays a key role in regulating glucose homeostasis in mice fed a high-fat diet (HFD). We found that there was sexual dimorphism in HCAR1 expression in BAT from mice fed HFD that may contribute to the development of hyperglycemia in male C57BL/6J DIO mice.

## Materials and methods

### Animals

All mouse care and experimental procedures were approved by the institutional animal care research advisory committee of the Albert Einstein College of Medicine. All experiments were performed in accordance with relevant guidelines and regulations. Mice used in experiments included C57BL/6J mice (The Jackson Laboratory, stock # 000664) and C57BL/6J DIO mice used as noted (the Jackson Laboratory, Stock # 380050). Both male and female mice were used for most experiments and were maintained with a 12 hours light-dark cycle. C57BL/6J mice at 5 weeks of age were fed a low fat diet (LFD, 70% calories provided by carbohydrates, 20% by protein, and 10% by fat; 3.85 kcal/g, D12492J, Research Diets), or a high fat diet (HFD, 20% calories by carbohydrate, 20% by protein, and 60% by fat; 5.21 kcal/g, D12492; Research Diets) with *ad libitum* access to water for 5 weeks at 28°C. Mice were euthanized by an overdose of Isoflurane.

### Measurement of body weight and blood glucose

During high-fat feeding, body weight was measured once a week at 9 AM. Following 5-week high-fat feeding at 28°C, mice were fasted overnight (from 7 PM to 9 AM) and fasting blood glucose levels were measured using a Contour blood glucose meter (Bayer, 7097C). To test the effect of the HCAR1 agonist on blood glucose levels, blood samples were collected from mouse tail at 0 and 4 hours post intraperitoneal (i.p.) injection of saline or 3,5-dihydroxybenzoic acid (3,5-DHBA, Tocris Bioscience) without overnight fasting. For these experiments, we used C57BL/6J DIO mice that were purchased from the Jackson laboratory.

### Measurement of 2-deoxy-D-glucose (2-DG) uptake

2-DG uptake by BAT was measured with a 2-DG uptake measurement kit (Cosmo bio co., ltd., CSR-OKP-PMG-K01TE) according to the manufacturer’s instruction. Mice received an i.p. injection of 2-DG (32.8 ug/kg [24], Fisher Scientific, AC111980050) 3 hr post i.p. injection of saline or 3,5-DHBA. BAT samples (10 mg) were isolated 1 hr post i.p. injection of 2-DG, and then homogenized in 10 mM Tris-HCl (pH8.1) on ice. Optical density of samples was measured at a wavelength of 420 nm using a microplate reader.

### Quantitative Real-time PCR analysis

For qRT-PCR analysis of monocarboxylate transporter 1 (*Slc16a1* (*Mct1*)), lactate dehydrogenase A (*Ldha)*, and hydrocarboxylic acid receptor 1 (*Hcar1* or *Gpr81*) genes, total RNA was isolated using the Trizol reagent (Thermo Fisher Scientific, Inc., 15596026) from BAT and then first-strand cDNAs were synthesized using RT Master Mix (Toyobo co., ltd, FSQ-201). Real-time qPCR was performed in sealed 96-well plates with SYBR qPCR master Mix (Toyobo co., ltd, QPS- 201). qPCR reactions were prepared in a final volume of 20 μl containing 2 μl cDNA and 10 μl of SYBR qPCR master mix in the presence of primers at 0.5 μM. TATA box binding protein (*Tbp*) was used as an internal control for quantification of each sample. A list of primer sets included: F5′-aatgctgccctgtcctccta-3′ and R5′-cccagtacgtgtatttgtag-3′ for *Slc16a1*, F5′-tcgtgcactagcggtctcaa-3′ and R5′-aacagcaccaaccccaaca-3′ for *Ldha*, F5′-tttgccagaggtgttgaagc-3′ and R5′-ggatactcaggttggtggct-3′ for *Hcar1*, and F5′-ccccttgtacccttcaccaat-3′ and R5′-gaagctgcggtacaattccaga-3′ for *Tbp*. Relative gene expression was determined using the *ΔΔCt* method. Relative mRNA expression levels were presented as a fold change compared with those of the control group.

### Western blotting

Whole cell lysates were prepared from BAT using lysis buffer (Sigma-Aldrich, C3228) with 1% protease inhibitor. Total lysates (20 μg each) were loaded in 12% SDS polyacrylamide gels and transferred to the PVDF membrane (Thermo Fisher Scientific, Inc., 88518). The membrane was incubated with 5% w/v nonfat dry milk for 2 hr at room temperature and then with anti-MCT1 (1:1,000, Novus Biologicals, NBP1–59656), anti-LDHA (1:1,000, Novus Biologicals, NBP1-48336), anti-HCAR1 (1:500, Sigma-Aldrich, SAB1300790), and anti-β-actin (1:5,000, Sigma-Aldrich, A5316) antibodies in TBST buffer containing 5% bovine serum albumin for overnight at 4°C. Following incubation with primary antibodies, the membrane was washed in TBST buffer, and then incubated with anti-mouse IgG, HRP-linked antibody (1:2,000, Cell Signaling Technology, Inc., 7076) or anti-Rabbit IgG, HRP-linked antibody (1:2,000, Cell Signaling Technology, Inc., 7074) for 2 hr at room temperature. All membranes were washed in TBST buffer, incubated with chemiluminescent substrate (Thermo Fisher Scientific Inc., 32109), and exposed to X-ray film (Thermo Fisher Scientific Inc., 34091). The relative band intensity was measured using ImageJ software.

### Statistics

All statistics were performed with GraphPad Prism software. Data were expressed as mean ± standard error (SEM). Statistical significance was determined using unpaired t-test or one-way ANOVA with turkey’s multiple comparison test (GraphPad Prism 7.0). Results with p < 0.05 were considered statistically significant.

## Results

### HCAR1 expression in C57BL/6J mice fed HFD

**Fig 1:**
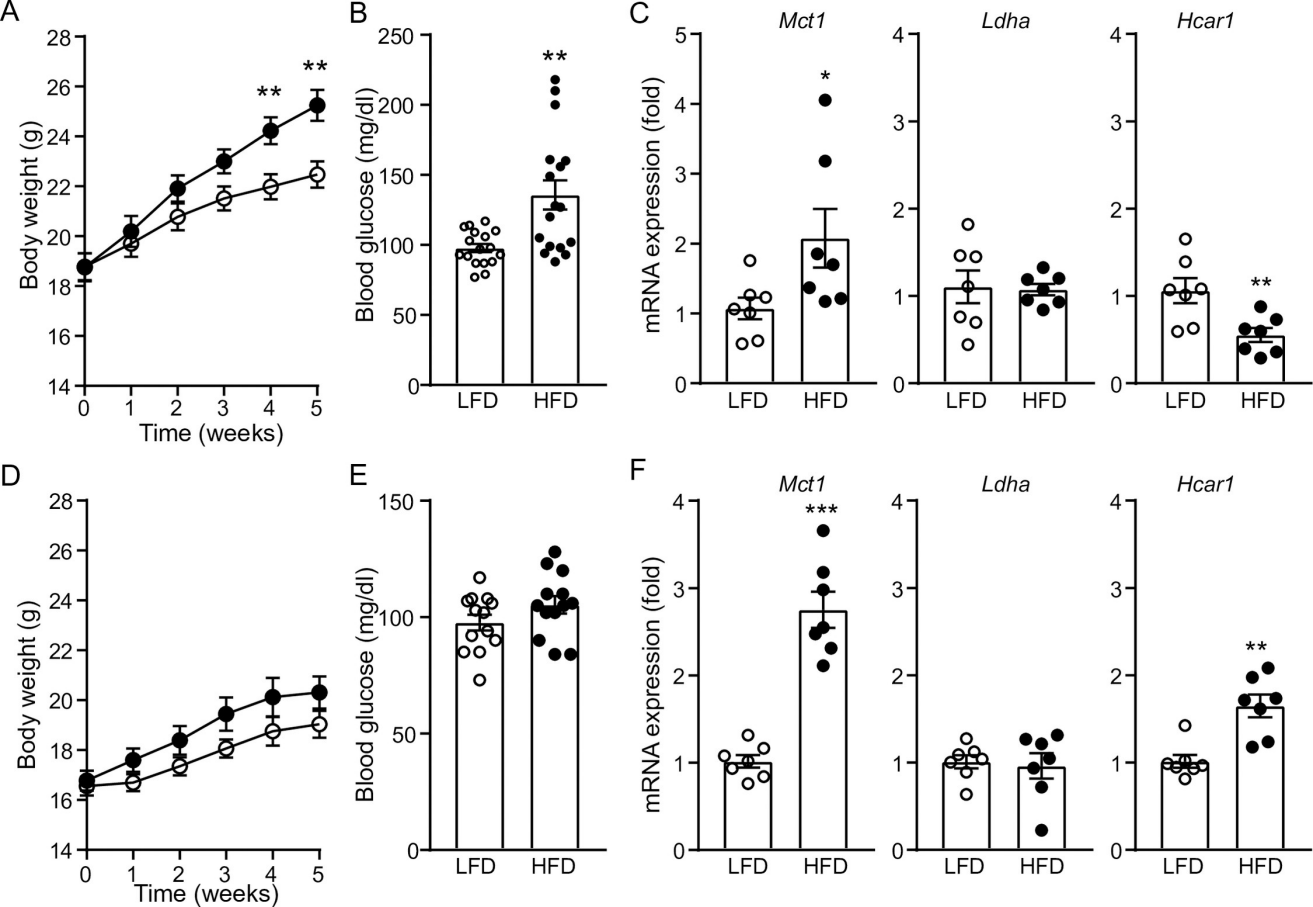
High-fat feeding differentially regulates *Hcar1* gene expression in male and female mice. **A** and **B.** Summary graphs showing changes in body weight and fasting glucose levels in male C57BL/6J mice fed LFD (open circle) and those on HFD (filled circle). Male mice fed HFD for 5 weeks at 28°C developed DIO and hyperglycemia (mean fasting blood glucose concentrations: LFD, 97.8 ± 3.0 mg/dl, HFD, 135.8 ± 10.4 mg/dl, n = 17 mice, respectively, **p<0.01). **C**. Summary plot showing altered expression of the genes required for lactate release and its cognate receptor in male mice fed HFD for 5 weeks (n = 7 and 7 mice / group, respectively, *p<0.05, **p<0.01). **D** and **E**. There were no differences in body weight and blood glucose levels in female mice fed either LFD or HFD for 5 weeks (mean fasting blood glucose concentrations: LFD, 97.7 ± 3.4 mg/dl, HFD, 105.3 ± 3.8 mg/dl, LFD, n = 13 mice; HFD, n = 13 mice). **F**. Summary plot showing altered expression of the genes required for lactate release and its cognate receptor in female mice fed HFD for 5 weeks. High-fat feeding significantly upregulated *Hcar1* mRNA expression (n = 7 and 7 mice / group, **p<0.01, ***p<0.001).

We recently showed that inhibition of LDHA blocked the ability of BAT to take up glucose [10], suggesting that lactate production in BAT is necessary for glucose uptake. Thus, we examined whether disrupted lactate production and signaling in BAT can cause hyperglycemia in mice fed HFD. Animals were kept at 28°C to limit cold stress and fed either LFD or HFD for 5 weeks. C57BL/6J male mice fed HFD gained more body weight than control mice fed LFD (Fig 1A) and developed hyperglycemia (Fig 1B). In contrast, there were no significant differences in body weight and blood glucose levels between female mice fed LFD and those on HFD (Fig 1D and 1E), consistent with prior studies showing that male C57BL/6J mice are more likely to develop DIO and hyperglycemia than female mice [25–28].

We next asked whether high-fat feeding alters expression of the genes required for lactate production, release, and signaling, including *Slc16a1* (*Mct1)*, *Ldha*, and *Hcar1*. Both male and female mice on HFD for 5 weeks at 28°C showed a significant increase in *Mct1* gene expression compared to those on LFD (Fig 1C and 1F). In contrast to higher levels of *Mct1* expression in mice fed HFD than those in mice fed LFD, there was no significant difference in *Ldha* expression in male and female mice following high-fat feeding (Fig 1C and 1F). Interestingly, high-fat feeding differentially regulated *Hcar1* gene expression in male and female mice. High-fat feeding significantly reduced *Hcar1* gene expression in BAT of male mice, while increasing its expression in BAT of female mice (Fig 1C and 1F). Our findings were consistent with the prior findings showing reduced *Hcar1* gene expression in adipose tissue of DIO male and *ob/ob* mice [29, 30].

**Fig 2:**
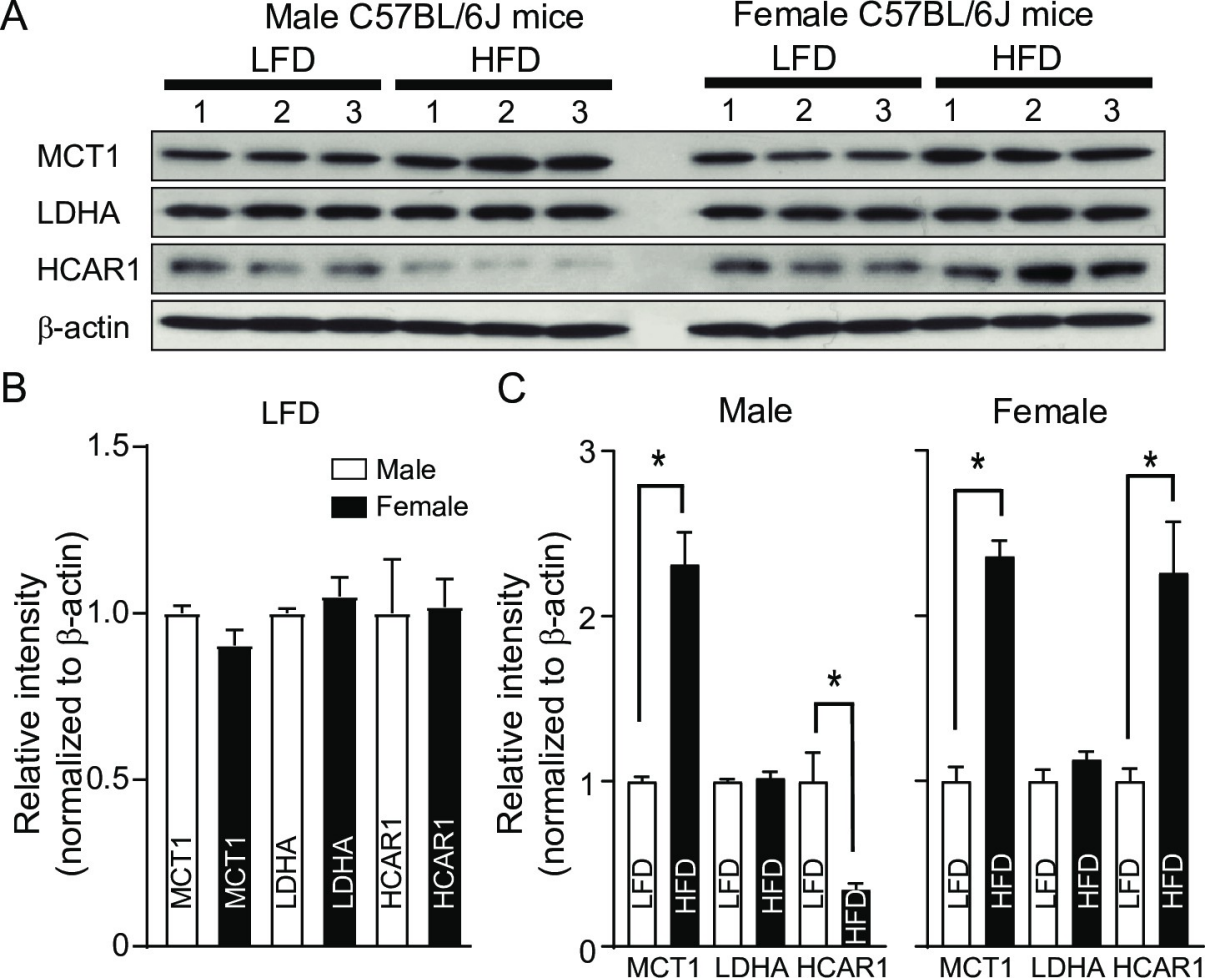
High-fat feeding differentially regulates HCAR1 protein expression in male and female mice. **A**. Images of Western blot of MCT1, LDHA, and HCAR1 in BAT from mice fed LFD or HFD for 5 weeks at 28°C (n = 3 mice/group/sex). **B.** Summary plot showing expression of MCT1, LDHA, and HCAR1 in male and female mice fed LFD for 5 weeks at 28°C. There were no differences in the expression of MCT1, LDHA, and HCAR1 (open bar: males, filled bar: females). **C.** Summary plot showing expression of MCT1, LDHA, and HCAR1 in male and female mice fed LFD or HFD for 5 weeks at 28°C (open bar: mice fed LFD, filled bar: mice fed HFD). High-fat feeding differentially regulated HCAR1 expression in male and female mice. *p<0.05.

We then performed Western blot analysis with anti-MCT1, anti-LDHA, and anti-HCAR1 antibodies. There were no differences in protein expression levels of MCT1, LDHA, and HCAR1 in BAT between male and female mice fed LFD (Fig 2B). In contrast, high-fat feeding oppositely regulated HCAR1 expression in male and female mice (Fig 2A and 2C). Similar to changes in the gene expression, there was a significant reduction in HCAR1 protein expression in male mice fed HFD, whereas female mice on HFD had an increase in HCAR1 expression. Additionally, MCT1 expression was upregulated in both male and female mice fed HFD (Fig 2A and 2B).

### Activation of HCAR1 lowers blood glucose through increased glucose uptake by BAT in male DIO mice

**Fig 3:**
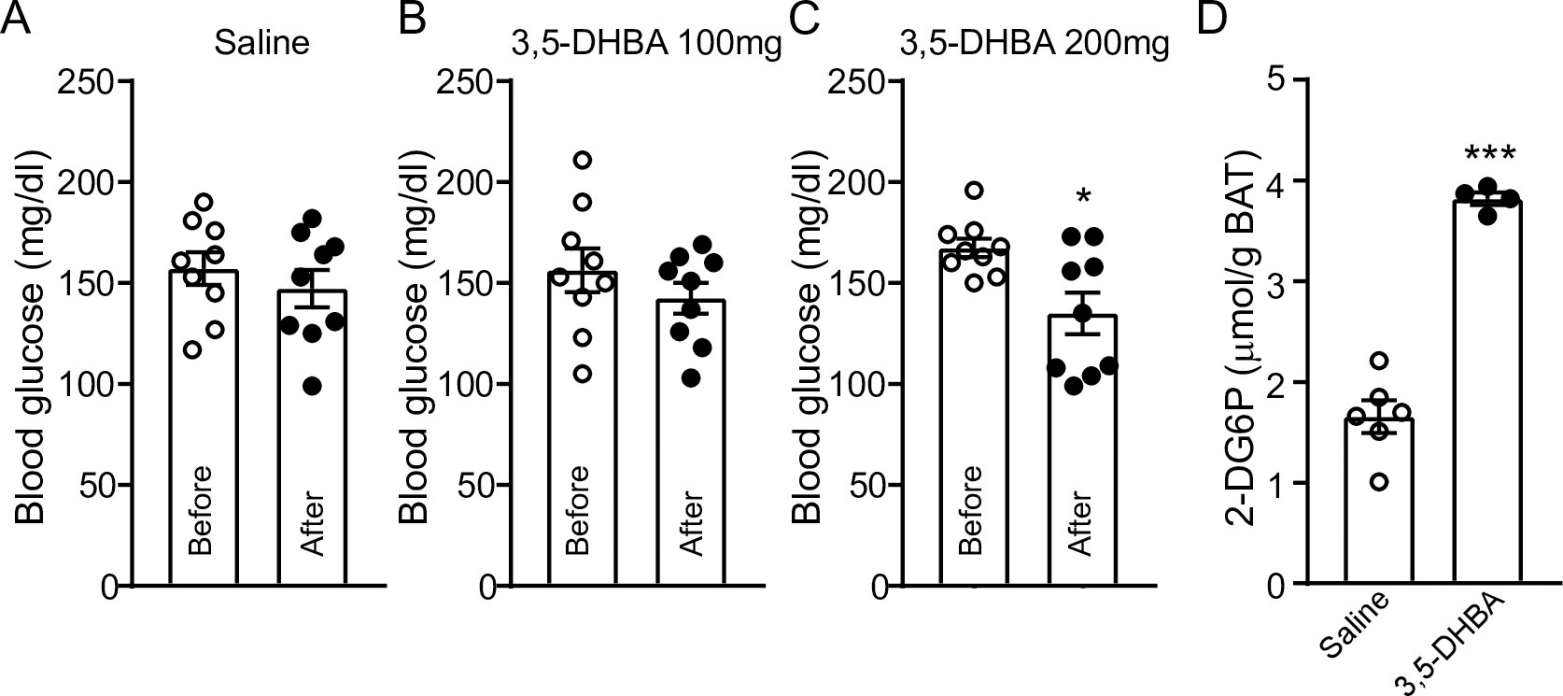
Acute effect of the HCAR1 agonist on blood glucose levels. **A-C**. Summary plot showing effect of 3,5-DHBA (0, 100, and 200 mg/kg) on blood glucose concentrations (n = 9 mice, respectively) (before: before i.p. injection of 3,5-DHBA, after: 4 hr after i.p. injection of 3,5-DHBA). Male DOI mice that were purchased from the Jackson lab were used for these experiments. * p < 0.05 **D**. Plot showing effect of 3,5-DHBA (200 mg/kg) on 2-DG uptake by BAT of male DIO mice (n = 6 and 4 mice, respectively). *** p < 0.001.

It has been shown that the antilipolytic effect of lactate was mediated through activation of HCAR1 in adipocytes [19, 20]. Insulin inhibited lipolysis through degradation of cAMP [31] and promoted glucose uptake through increased glucose transporter 4 exocytosis in brown adipocytes [32, 33]. We asked whether activation of HCAR1 can induce glucose uptake, resulting in a reduction in systemic glucose concentrations in male DIO mice. To activate the HCAR1, the HCAR1 agonist 3,5-dihydroxybenzoic acid (3,5-DHBA; 100 and 200 mg/kg [34, 35]) was intraperitoneally injected to male DIO mice. Following i.p. injection of 3,5-DHBA or saline to male DIO mice, we measured blood glucose at 0 and 4 hr (Fig 3A). We found that treatment with 3,5-DHBA (200 mg/kg) significantly reduced blood glucose levels in male DIO mice 4 hr post treatment, supporting the interpretation that HCAR1 activation lowers blood glucose levels in male DIO mice.

We further examined whether this reduction is due in part to improved glucose uptake by BAT. We measured 2-deoxy-D-glucose (2‐DG) uptake by BAT from mice treated with 3,5-DHBA (200 mg/kg, n = 4 mice) or saline (n = 6 mice). Direct measurement of 2-DG-6-phosphate (2-DG6P) amount accumulated in BAT revealed that treatment with 3,5-DHBA significantly increased the accumulation of 2-DG6P (Fig 3B), indicating that activation of HCAR1 in BAT promotes glucose uptake by BAT.

## Discussion

We provide physiological evidence that HCAR1 in BAT plays an important role in the control of glucose homeostasis in mice. We found that high-fat feeding downregulated HCAR1 expression in BAT of male C57BL/6J mice. This impairment was associated with hyperglycemia in male mice fed HFD. Importantly, treatment with the HCAR1 agonist was able to restore blood glucose concentrations in part through improved glucose uptake by BAT in DIO male mice. In contrast, female C57BL/6J mice did not develop hyperglycemia during high-fat feeding. High-fat feeding upregulated HCAR1 expression in BAT. Although it remains to be determined whether HCAR1 expressed in other peripheral tissues such as white adipocytes and skeletal muscle [19, 20] can contribute to the control of systemic blood glucose levels, our present study suggest that impaired HCAR1 function may cause metabolic dysfunction in BAT and consequently disrupt glucose homeostasis.

It has been described that there is sexual dimorphism in BAT morphology in rodents [36]. In particular, female rats have larger mitochondria with higher cristae density and UCP1 content than male rats, indicating that female rats have an increased thermogenic activity in BAT [36]. In humans, female individuals also have a greater mass of brown adipose tissue and higher BAT activity than male subjects [37, 38]. In addition, proteomic analysis of rodent BAT shows differential regulation of BAT proteins in response to high-fat feeding [39]. For instance, expression of fatty acid synthase (FAS) is downregulated in female rats fed HFD, while high-fat feeding increases its expression in male animals [39]. Interestingly, UCP1 mRNA expression is higher in females compared with male mice fed HFD for 28 days [40]. Male mice fed HFD gain significantly more weight than controls, whereas a significant increase in body weight is observed only after 60 days of high-fat feeding in female mice [40]. Our present results further revealed that high-fat feeding oppositely regulated HCAR1 expression in BAT of female and male C57BL/6J mice. Male mice exhibited reduced HCAR1 expression in BAT, which may result in higher lipolysis in male mice than in female mice. As obesity is strongly associated with increased lipolysis in adipocytes [41, 42], reduced HCAR1 expression and as a result, increased lipolysis in male DIO mice can cause hyperglycemia. Therefore, it is likely that sexual dimorphism in BAT morphology, proteome, and responses to overfeeding may play a critical role in the development of DIO and metabolic diseases.

BAT is considered a secretory organ and releases several endocrine factors, including fibroblast growth factor 21, slit2-C, and neuregulin 4 [43]. A recent study of human BAT further demonstrates that BAT, but not WAT, continuously releases lactate in warm conditions [6]. An autocrine and/or paracrine release of lactate blocked lipolysis in adipocytes through activation of HCAR1 [20]. These prior studies suggest that lactate may act as a signaling molecule in BAT. Similar to insulin’s actions in BAT, HCAR1-medaited inhibition of lipolysis in BAT may promote glucose uptake by BAT. Indeed, treatment with the HCAR1 agonist readily induced 2-DG uptake in BAT of DIO male mice in our preparations. In addition, our prior study showed that lactate production in BAT was required for β3-adrenergic receptor-mediated glucose uptake [10]. Therefore, lactate production and its cognate receptor activation may improve the capability of BAT to take up circulating glucose in warm and cold conditions.

Our present findings are consistent with the fact that lactate acts as an important signaling molecule in the control of overall glucose homeostasis. In fact, increased body weight and hyperglycemia were associated with reduced expression of HCAR1 in BAT of male C57BL/6J mice fed HFD, consistent with reduced HCAR1 expression in adipose tissue of DIO male and *ob/ob* mice [29, 30]. In contrast, upregulated HCAR1 expression in BAT from female C57BL/6J mice fed HFD appears to prevent hyperglycemia and body weight gain. As basal lipolysis in adipocytes was elevated during obesity and was closely associated with insulin resistance [41, 42], inhibition of lipolysis through activation of HCAR1 in adipose tissue BAT would be an alternative way to control glycemia in individual with type 2 diabetes. In fact, patients with type 2 diabetes show reduced glucose uptake by BAT, although fatty acid uptake and metabolism are not defective in type 2 diabetes [14]. Moreover, glucose uptake rates by BAT are lower in response to the β3AR agonist in mice fed HFD than in mice fed a regular chow [44]. However, HCAR1 is also expressed in white adipocytes and skeletal muscle [19, 45] and activation of HCAR1 blocks lipolysis or induces triglyceride accumulation in these tissues [19, 20, 35, 45]. Thus, we cannot rule out the involvement of HCAR1 in other peripheral tissues in the control of glucose homeostasis. Interestingly, high-fat feeding for 11 weeks reduces HCAR1 gene expression in white adipose tissue of male C57BL/6 mice [29]. Decreased expression of HCAR1 in white as well as brown adipose tissue of male mice may result in hyperglycemia in obese male animals.

The cellular mechanisms that mediate glucose uptake by BAT upon activation of HCAR1 remain to be determined in cell preparations. Interestingly, both lactate and insulin reduce cAMP levels by either inhibiting cAMP formation or promoting cAMP degradation, resulting in inhibition of lipolysis[18]. It is plausible that, as like insulin, activation of HCAR1 by lactate can induce glucose transporter 4 (GLUT4) translocation in BAT. In fact, HCAR1 activates phospholipase C (PLC) through the Gβγ subunit [46]. PLC has been shown to be involved in insulin-induced translocation of GLUT4 [47, 48]. Alternatively, as described in the study of Ahmed and colleagues [20], HCAR1 may improve insulin’s actions in adipose tissue. As the activation of HCAR1 in BAT induces antilipolytic and antidiabetic effects, our findings have potential to lead to the discovery of novel therapeutic targets for better treatment of type 2 diabetes.

## Supporting information

S1 Fig Image of western blotting showing expression of MCT1, LDHA, HCAR1, and β-actin. (PDF)
