# Supplementary material for: Hydrocarboxylic acid receptor 1 in BAT regulates glucose uptake in mice fed a high-fat diet
Source: PLoS One. 2020 Jan 30;15(1):e0228320. doi: 10.1371/journal.pone.0228320 (PMC6992197; doi:10.1371/journal.pone.0228320)
Supplement: S1 Fig — (PDF) [file pone.0228320.s001.pdf]

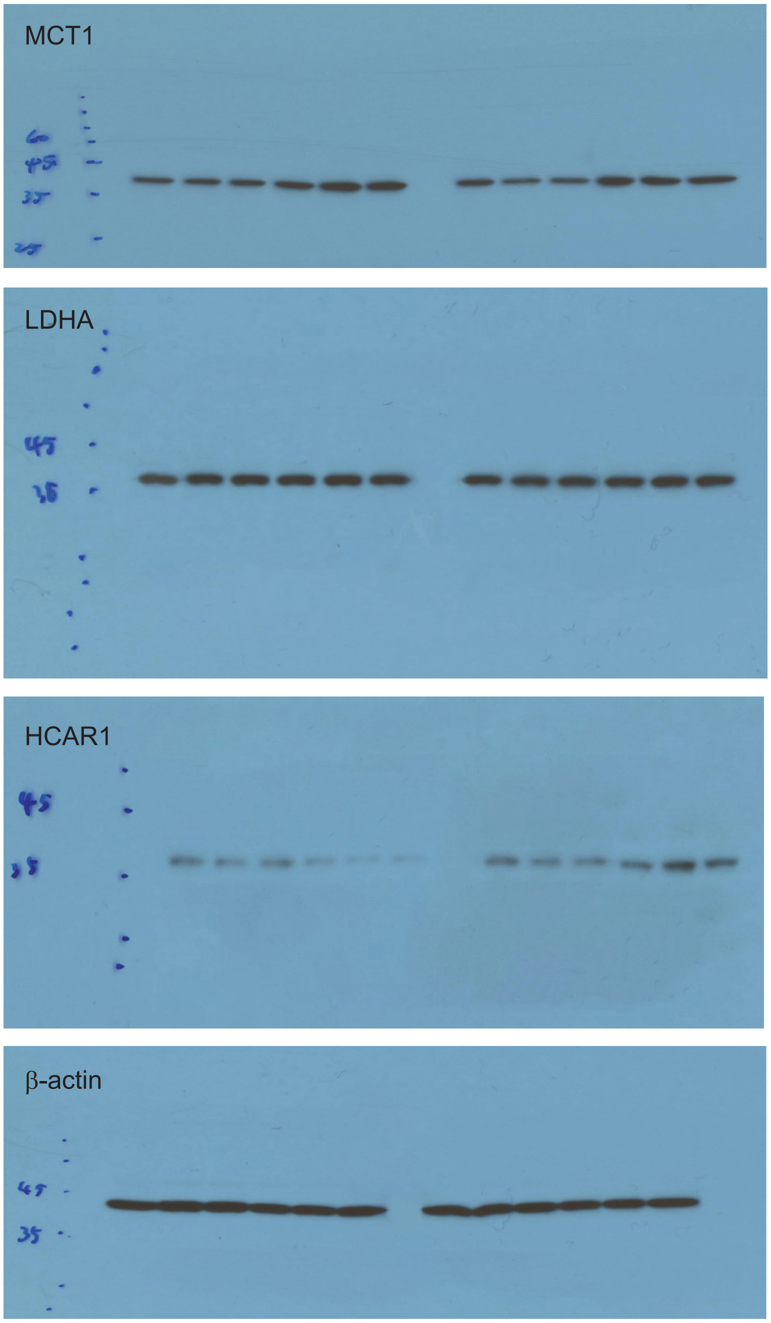

Supplementary Figure 1. Image of Western Blotting showing expression of MCT1, LDHA, HCAR1, and β-actin
